# Key MicroRNA’s and Their Targetome in Adrenocortical Cancer

**DOI:** 10.3390/cancers12082198

**Published:** 2020-08-06

**Authors:** Marthe Chehade, Martyn Bullock, Anthony Glover, Gyorgy Hutvagner, Stan Sidhu

**Affiliations:** 1 Cancer Genetics Laboratory, Kolling Institute, Northern Sydney Local Health District, St. Leonards, NSW 2065, Australia; mche2952@uni.sydney.edu.au (M.C.); martyn.bullock@sydney.edu.au (M.B.); anthony.glover@sydney.edu.au (A.G.); 2 Sydney Medical School Northern, Royal North Shore Hospital, University of Sydney, Sydney, NSW 2065, Australia; 3 Endocrine Surgery Unit, Royal North Shore Hospital, Northern Clinical School, Faculty of Medicine and Health, The University of Sydney, St. Leonards, Sydney, NSW 2007, Australia; 4 School of Biomedical Engineering, Faculty of Engineering and Information Technology, University of Technology Sydney, Sydney, NSW 2007, Australia

**Keywords:** adrenocortical carcinoma, micro RNA, non-coding RNA

## Abstract

Adrenocortical Carcinoma (ACC) is a rare but aggressive malignancy with poor prognosis and limited response to available systemic therapies. Although complete surgical resection gives the best chance for long-term survival, ACC has a two-year recurrence rate of 50%, which poses a therapeutic challenge. High throughput analyses focused on characterizing the molecular signature of ACC have revealed specific micro-RNAs (miRNAs) that are associated with aggressive tumor phenotypes. MiRNAs are small non-coding RNA molecules that regulate gene expression by inhibiting mRNA translation or degrading mRNA transcripts and have been generally implicated in carcinogenesis. This review summarizes the current insights into dysregulated miRNAs in ACC tumorigenesis, their known functions, and specific targetomes. In addition, we explore the possibility of particular miRNAs to be exploited as clinical biomarkers in ACC and as potential therapeutics.

## 1. Introduction

Adrenal tumors are very common, affecting up to 10% of the general population, of which the large majority are benign non-functional adenomas [1]. Adrenocortical cancer (ACC), in contrast, is a rare endocrine malignancy with an incidence of 0.76 per million in the general population [2]. Approximately 60% of patients with ACC present with signs and symptoms of hormone excess [3], and approximately 20% present with mass associated symptoms, such as abdominal pain, early satiety, or abdominal fullness [4]. The remaining patients are incidentally diagnosed on abdominal imaging for other medical indications. As the clinical manifestations of hormone excess may be subtle and mass effect symptoms are vague, ACC is often diagnosed late. The median size of the primary tumor is 12 cm at diagnosis [5], and the rate of unresectable metastatic disease at diagnosis ranges between 30% [6] and 70% [7]. Complete surgical resection with oncologically clear margins affords the best chance of cure in ACC but even despite this, the rate of disease recurrence is high, and the prognosis is generally poor with five-year survival of less than 40% [8].

Management options for metastatic ACC are limited as cytotoxic chemotherapy affords only a marginal survival benefit, and mitotane, an adrenolytic agent, which is the only other approved systemic therapy for metastatic ACC, is poorly tolerated. The first and only randomized controlled chemotherapy-based phase III clinical trial for advanced ACC (First International Randomized Trial in Locally Advanced and Metastatic Adrenocortical Carcinoma Treatment (FIRM-ACT)) was completed in 2010. This study compared mitotane administered in combination with either streptozocin or etoposide, doxorubicin, and cisplatin (EDPM) and demonstrated a modest improvement in progression-free survival in the EDPM arm, but no benefit in the overall survival [9]. ACC is, therefore, an orphan disease that presents challenges on both diagnostic and management fronts. ACC research is currently focused on developing methods for early detection and effective management of a metastatic disease. In particular, the discovery of novel approaches to the management of metastatic ACC is crucial to improving patient outlook.

MicroRNA (miRNA) are small non-protein-coding RNA molecules whose deregulation has been implicated in the pathogenesis of many human diseases, particularly cancer. Over the past two decades, miRNA research in cancer has focused on determining the miRNA expression signatures of different tumors in order to identify potential biomarkers for early diagnosis, as well as functional studies of specific miRNAs to determine their targets and function. The set of mRNAs targeted by a defined miRNA is known as its targetome. While multiple studies have profiled the miRNA signature of childhood and adult ACCs using various techniques and shown consistent deregulation in a set of candidate miRNAs, relatively fewer have demonstrated miRNA-target interactions. MiRNAs have also been identified to have both diagnostic and therapeutic potential in the cancer literature, broadening our understanding of their roles in tumor biology. In this review, we present a current summary of the mounting body of work describing miRNA dysfunction in ACC with the aims of highlighting their potential function and roles in modulating key oncogenic pathways.

## 2. ACC Genetic Landscape and Associated Genetic Disorders

ACC has a bimodal distribution with a worldwide childhood incidence of 0.2 per million [10], and an adult peak in the fifth decade of life. Childhood ACC differs from adult ACC, as 50–80% of childhood cases are associated with germline *TP53* mutations [11,12]. In contrast, most cases of adult ACC are sporadic, with germline *TP53* mutations being present in around three percent of patients [13].

In rare cases, ACC can be associated with specific germline mutations that cause hereditary cancer syndromes. ACC is a core malignancy in Li Fraumeni Syndrome (LFS) caused by the germline *TP53* mutation and affects ten percent of cases [14]. Notably, in a study by Soon and colleagues, sporadic ACC was associated with loss of heterozygosity (LOH) at the *TP53* gene locus 17p13.1 in 74% of cases compared with only 14% of adrenal adenomas [15]. ACC affects approximately seven percent of children with Beckwith–Weidemann Syndrome (BWS) [16], which is caused by mutations or epigenetic modifications at the genetic locus 11p15 containing the *Insulin-Like Growth Factor 2* (*IGF2*) gene. 11p15 LOH or *IGF2* overexpression were demonstrated in 93.1% of sporadic ACCs compared with only 8.6% of benign adrenal tumors [17] in a study by Gicquel and colleagues, highlighting the importance of this imprinted locus in the pathogenesis of ACC. Approximately three percent of patients with Familial Adenomatous Polyposis (FAP), caused by mutations in the Adenomatous Polyposis Coli (APC) gene, develop adrenocortical cancer as adults [18]. The APC protein is a negative regulator of β-Catenin, whose accumulation in the nuclei of primary ACCs has been associated with advanced tumor stage and poor prognosis [19]. ACC is also rarely associated with Lynch Syndrome, Neurofibromatosis Type 1, and Carney Complex, as well as Multiple Endocrine Neoplasia Type 1 (MEN1) in adults [20] (Table 1). Although mutations in *TP53*, *IGF-2*, and *β-catenin* genes have been established as drivers of sporadic ACC, the low penetrance of ACC in these genetic cancer syndromes indicates that mutations or the epigenetic regulation of the expression of nearby genes may play an important role in its etiology.

## 3. Key Genetic Drivers of ACC and Their Cellular Pathways

It is now known that mutations in gene drivers alone do not completely explain the pathogenesis of ACC, and therefore non-coding gene mutations that lead to aberrant regulation of driver genes through their pathways can also contribute to tumor biogenesis. The following summary of key genetic drivers in ACC, therefore, serves to explore the extent to which ACC pathogenesis could be explained by them, and contextualize the importance of known miRNA targets within these pathways.

### 3.1. Tumor Suppressor Protein 53 (TP53)

The *TP53* gene encodes a homo-tetrameric transcription factor that mediates the cellular response to genotoxic stress and the activation of oncogenes by transcriptionally targeting many genes to ultimately activate cellular pathways involved in cell-cycle arrest and DNA damage repair. Where the cell fails to repair this damage, p53 induces cellular apoptosis via a p53-upregulated modulator of apoptosis (PUMA) to avoid propagating genetic mistakes. P53 is regulated by Human Double Minute 2 homolog (HDM2), an E3 ubiquitin-protein ligase, which targets p53 for cytosolic translocation, or proteosomal degradation when it is polyubiquitinated. HDM2 is, in turn, regulated by p53, forming a negative feedback loop [26].

*TP53* mutations in cancer are common and are present in more than half of human tumors [27]. ACC, despite its rarity, accounts for 11.9% of all human tumors harboring germline *TP53* mutations, after breast, soft-tissue, and brain tumors [28]. ACC’s harboring somatic TP53 mutations are on average larger, more advanced in stage, and associated with shorter disease-free survival [29]. The majority of *TP53* mutations associated with ACC are loss-of-function mutations; however, many of these are predicted to result only in partial loss of p53 function [30]. Curiously, transgenic *TP53* knockout and mutant mouse models do not develop ACC despite developing multiple tumors [31,32]. Else and colleagues showed that transgenic mice carrying an inactivation mutation in the *tripeptidyl peptidase 1/ACD sheltering complex subunit and telomerase recruitment factor* (*Tpp/Acd*) in addition to a single wild type *TP53* allele do develop ACCs at low frequencies [33].

### 3.2. Insulin-Like Growth Factor 2 (IGF2)

IGF2 is a paternally imprinted critical growth factor in the development of many organ systems, including the adrenal cortex, where it is highly expressed in early fetal development [34]. Multiple studies have confirmed *IGF2* overexpression in between 83.3% and 90.9% of ACC’s when compared with ACA and NAC [35,36,37,38]. The maternally imprinted long non-coding RNA *H19* gene located on the antisense strand of the *IGF2* gene is shown to be underexpressed in ACC compared with ACA and NAC in multiple studies [39,40,41,42]. Both LOH [17] and paternal uniparental disomy at the 11p15 locus result in *IGF2* overexpression and reduced expression of *H19* and *Cyclin-dependent kinase inhibitor 1C* (*CDKN1C*) in ACC [36], which are associated with poor prognosis and increased rates of recurrence [43].

IGF2 binds to the membrane tyrosine kinase receptor IGF Receptor Type 1 (IGF-1R), leading to receptor autophosphorylation and binding of the insulin receptor substrate 1 (IRS-1). Tyrosine phosphorylation of IRS-1 activates the phosphatidylinositol-3-kinase(PI3K)/serine/threonine protein kinase B (Akt) and mammalian target of rapamycin(mTOR) pathway as well as the Ras/Raf/mitogen-activated protein kinase (MEK)/extracellular signal-related kinase (ERK) pathways, potentiating cellular proliferation and viability in ACC cell models [44].

Transgenic mouse models of *IGF2* overexpression [45] and adrenal cortex-specific loss of imprinting at the *IGF2/H19* region [46] have demonstrated that these factors alone are not sufficient to initiate tumorigenesis. From a therapeutic approach, clinical trials of the IGF-1R small molecule inhibitors, Linsitinib and Figitumumab to treat advanced ACC failed to show benefit in progression-free survival or overall survival [47,48]. Another trial involving 26 ACC patients treated with the IGF-1R antibody Cixutumumab in combination with the mTOR inhibitor Temsirolimus achieved stable disease for at least six months in 42% of patients but did not lead to any partial or complete responses to therapy [49].

### 3.3. Wnt/β-Catenin Signalling Pathway

The Wnt signaling pathways are activated by Wnt-protein ligand binding extracellularly to a membrane Frizzled receptor. Canonical Wnt pathway activation leads to the accumulation of β-catenin in the cytoplasm, which ultimately translocates to the nucleus where it activates transcription. In the absence of Wnt, β-catenin is degraded by a protein complex formed by Axin, APC, protein phosphatase 2A (PP2A), glycogen synthase kinase 3 (GSK3) and casein kinase 1α (CK1α), by targeting it for ubiquitination and ultimate proteosomal degradation [50].

Assie and colleagues performed exome sequencing and single nucleotide polymorphism (SNP) analysis of 77 ACC tissues and showed alterations in the β-catenin pathway associated genes *zinc and ring finger 3* (*ZNRF3*) (21%), *cadherin-associated protein β1* (*CTNNB1*) (16%) and *APC* (2%) [51]. More strikingly, in a series of 50 ACC tissues, Maharjan and colleagues reported that the Wnt/β-catenin pathway was aberrantly activated in 62% [52].

Transgenic mouse models of constitutive *β-catenin* activation in the adrenal cortex produced aggressive adrenal tumors only in a subset of 17-month-old mice [53], while *APC* knockout mice displayed hyperplasia progressing to adrenal adenoma but not carcinoma [46].

Together these findings indicate that multiple genetic aberrations are required for the development of ACC, and combinatorial therapeutic strategies targeting multiple pathways may be effective.

## 4. Overview of microRNA Structure, Biogenesis, and Function

MiRNAs are short, single-stranded non-coding RNA molecules spanning between 17–25 nucleotides. Approximately 2,300 miRNAs have been identified in the human genome [54]. Their expression is tissue-specific, and they broadly act to negatively regulate the gene expression of at least 60% of human RNA transcripts through either translational inhibition or transcript decay [55]. While the majority of miRNA targets are mRNAs, other classes of RNA, including rRNA, tRNA, lncRNA, and other miRNAs make up 30% of all miRNA targets [56].

The RNAse enzymes DICER and DROSHA involved in miRNA maturation have been implicated in ACC. In a study which compared the expression of key miRNA processing factors between 29 ACC and 43 adrenocortical adenoma (ACA) tissues, Caramuta and colleagues showed that DICER, DROSHA, and TAR RNA-binding protein 2 (TARBP2) (a DICER cofactor required for miRNA processing [57]) were overexpressed at the mRNA and protein levels in ACC compared with ACA [58]. In addition, in vitro inhibition of *TARBP2* expression in the ACC cell line H295R resulted in decreased cellular proliferation and increased apoptosis [58]. This evidence suggests that dysregulation of the miRNA biogenesis pathway may potentiate tumorigenesis in ACC.

In the cytoplasm, the mature miRNA duplex unwinds from the thermodynamically less stable end, and the RNA strand that orients its 5′ end in this direction known as the guide strand is loaded onto the RNA-induced silencing complex (RISC) [59]. RISC is a multiprotein complex containing one member of the Argonaute protein family [60]. miRNAs contain a seed region spanning 2–8 nucleotides at their 5’ ends, which allows them to guide RISC mainly to the 3´ untranslated region (UTR) of their target mRNA through complementary base pairing. The degree of complementarity between miRNA and mRNA and the enzymatic properties of the Ago-2 protein determine whether mRNA silencing will be achieved through target cleavage or translational inhibition [61].

In cancer, the dysregulation of miRNA expression results from various mechanisms, including amplification or deletion of miRNA genes, dysregulation of transcriptional machinery, changes in methylation and histone modifications, as well as mutations and changes in expression of miRNA biogenesis-related proteins [62,63]. As miRNAs play an important role in the regulation of gene expression, their aberrant expression can lead to significant alterations in cellular phenotype. In their dysregulated state miRNAs can act as either oncogenes or tumor suppressors, affecting the cellular processes required for tumor initiation and progression.

### IsomiRs and Their Emerging Significance in Cancer

Inaccurate cleavage by either DICER or DROSHA, nucleotide additions at the 3´ end, and nucleotide modifications could result in the production of miRNA isoforms (isomiRs) [64]. Increasing data shows that isomiRs have significant impacts on miRNA-mediated gene regulation [65]. Variations in the 5´ seed sequence could impact the specificity of miRNAs to their targets [66], and variations at the 3´ ends determine the stability of miRNA-mRNA binding [67]. The impact of a particular isomiR on miRNA function depends on the relative abundance and stability of the isomiR relative to its canonical miRNA, as well as its binding efficiency to its target. Chan and colleagues demonstrated this by showing that isomiRs of *miR-31* regulated the expression of known targets to varying degrees *in vitro*, and their binding capacity to the RISC complex determined using Ago-2 immunoprecipitation (IP) was also varied [68].

Recent advances in high-throughput RNA sequencing technologies have allowed tissue transcriptome profiling at the isomiR level. Telonis and colleagues [69] used The Cancer Gene Atlas (TCGA) miRNA sequencing data across 32 cancer types, including ACC, to determine whether the presence or absence of isomiRs could discriminate between the cancer types. By binarizing the isomiR expression data, they were able to successfully classify tumor datasets with an average sensitivity of 90% and false discovery rate (FDR) of 3%, which was superior to wild type miRNA expression (average sensitivity 83%, FDR 5%) [69]. More recently, Wang and colleagues used the same data to demonstrate that isomiRs that share their seed region (5′ isoforms) could similarly discriminate between tumors [70]. In 2018, Lan and colleagues published the first study to use breast cancer TCGA small RNA sequencing expression data to show that the isomiR expression-based classification was superior to gene expression profiling at distinguishing between breast cancer subtypes [71]. Together these findings suggest that miRNAs may carry out significant functional roles in tumorigenesis at the isomiR level.

A study that investigated isomiR expression in adrenal tissues using RNA sequencing technology published in 2017 found that 411 miRNAs existed as 1763 various isoforms in a cohort of 14 ACC, 18 ACA, and 18 NAC samples [72]. These isomiRs contained 520 various seed sequences, of which 38% were non-canonical. Over a quarter of all expressed miRNAs in the ACC, ACA, and NAC groups produced isomiRs with two or more seed regions that were predicted to target a different set of mRNAs, but these were not investigated further. Therefore, the diagnostic and clinical significance of differentially expressed isomiRs in ACC remains unknown, and further research is needed to elucidate this.

While technical challenges related to the identification and quantification of isomiRs exist, recent developments in data sharing and technology are helping to overcome these. Often isomiR sequencing data is drawn from small sample numbers making it difficult to make general conclusions, but this is being overcome by the release of small RNA sequencing data from large sample databases such as TCGA. This has allowed researchers to study isomiR expression in more detail than previously possible. Also, recently developed specialized techniques such as photoactivatable ribonucleoside-enhanced crosslinking and Ago immunoprecipitation (Ago PAR-CLIP) ensures that identified isomiRs are biologically active and not degradation products, improving the reliability of the experimental data in this field. The utilization of such advances will facilitate research that will unlock a deeper understanding of the role of isomiRs in ACC.

## 5. The Unique microRNA Expression Signature of ACC and Its Clinical Significance

### 5.1. The microRNA Expression Signature of ACC Tissues

Sporadic ACC is a genetically heterogeneous malignancy that can be classified into distinct groups based on transcriptomics and clinical behavior. Several published studies have profiled differential miRNA expression in ACC tissue samples compared with either ACA and/or normal adrenal cortex (NAC) tissue using microarray data [73,74,75,76,77], TaqMan Low-Density Arrays (TLDA) [78,79], RT-q-PCR [80], or RNA sequencing [51,72,81] (Table 2). Across these studies, *miR-483-5p*, *miR-503-5p*, *miR-210*, and *miR-483-3p* were overexpressed in ACC compared with ACA or NAC in multiple datasets, and *miR-195*, *miR-497*, and *miR-335* were underexpressed. Of note, *miR-483-5p* was overexpressed in eight of eleven studies, and has been associated with poor prognosis in ACC [73]. Earlier studies using microarray and RT-q-PCR techniques could only investigate known miRNAs, whereas later studies which utilized RNA sequencing could identify differentially expressed miRNAs which had not previously been characterized. Hence, more numerous microRNA candidates have been identified with RNA sequencing, and novel candidates like *miR-508-3p* were only identified and validated in these later studies [51,81].

### 5.2. Circulating microRNAs as Diagnostic Biomarkers in ACC

Diagnosing patients with ACC continues to present challenges as no preoperative blood-borne tumor marker for the disease exists, and suspicion is often raised on imaging. MiRNAs are stable in bodily fluids within extracellular vesicles shed from tumor cells or in protein complexes and are relatively protected from enzymatic degradation in the circulation [82], making them attractive as potential noninvasive diagnostic markers for cancer. Despite a significant body of research confirming differentially expressed circulating miRNAs in various diseases, none have translated into clinical use.

A number of studies have reported on the expression and diagnostic utility of circulating miRNAs in ACC, whose findings have been summarized in a recent review by Decmann and colleagues [83]. Several of these studies have attempted to define candidate diagnostic circulating miRNA biomarkers for ACC in serum [76,84,85], but both relatively low sensitivity and specificity values have limited their clinical application. Although circulating *miR-483-5p* relative expression is a reliable differentiator between aggressive and non-aggressive ACC in serum, as demonstrated by Chabre and colleagues (AUC 0.929) [76], it is unable to differentiate between ACC and adrenocortical adenoma (AUC 0.74) [85]. Circulating *miR-483-5p* is also overexpressed in hepatocellular [86] as well as head and neck cancers [87], and has been proposed as a diagnostic marker in these diseases as in ACC. The lack of specificity of this miRNA as a biomarker further limits its clinical utility in ACC, and is characteristic of oncological miRNAs across different tumor types [88].

At present, various methods are in use for the quantification of circulating miRNAs in preclinical research. The lack of standardization in sample collection, storage, and processing introduces significant variation in the data, which limits the generalizability of differential expression results [89]. In addition, common reference RNA genes used as calibrators in comparing miRNA expressions are differentially expressed in the serum of patients with various diseases, leading to challenges in data normalization for analysis [90]. To overcome this, the addition of synthetic RNA during RNA extraction as a spike-in control is a method widely used for technical normalization. Such an exogenous control is helpful as it undergoes the same processing as endogenous RNA in the sample, but this does not correct for variables such as the serum miRNA fraction [91]. Standardization of sample collection techniques, developments in vesicle-associated miRNA quantification, and the use of absolute quantification methods that do not rely on housekeeper genes could help overcome the obstacles to the clinical adoption of circulating miRNAs as diagnostic biomarkers in the future.

### 5.3. Tissue microRNA Expression as a Prognostic Tool in ACC

In patients with ACC, the prescribed adjuvant clinical management and follow-up regimen is informed by the estimated risk of disease recurrence. Even in patients with unresectable disease, systemic therapy depends on tumor biology. While clinical and pathological prognostic indicators such as tumor stage, pathological grade, Ki67 proliferation index, and resection status are helpful in estimating survival, more recently, genomic and transcriptome based studies have identified molecular markers that can help predict recurrence-free survival as well [92]. Various RT-q-PCR studies have shown that tissue relative expression levels of *miR-210*, *miR-483-5p*, *miR-195*, *miR-503*, *miR-1202*, and *miR-1275* are associated with overall survival in ACC [73,74,80]. In addition, ACC tissue *miR-9* relative expression has been shown to correlate with recurrence-free survival as well as overall survival [93], as has the serum relative expression of *miR-483-5p* and *miR-195* [76].

In an RNA sequencing study of 45 ACC and three NAC tissues, Assie and colleagues used consensus clustering to classify tumors into three groups based on their microRNA profiles. The cluster most distinct from NAC, Mi1, was also characterized by consistent 14q32 LOH and *maternally expressed 3* (*MEG3*) long non-coding RNA promotor methylation. The 14q32 cytogenetic band contains 54 miRNAs, one of the largest miRNA clusters in the human genome, and 38 of these were underexpressed in the good prognosis of Mi1 tumors. The Mi2 group was characterized by weak overexpression of the *miR-506-514* cluster, known to have an oncogenic role in melanoma [94], while the Mi3 group was strongly correlated with the poor prognosis transcriptome cluster C1A. Interestingly, while miRNA expression was maximally deregulated in Mi1 and Mi2 cluster tumors, ACC driver pathway alterations were more consistently associated with Mi3 cluster tumors [51]. This study suggests that the integrated analysis of miRNA expression is likely to be a superior approach to single miRNA prognostic biomarkers for ACC.

## 6. Computational and Experimental Methods of miRNA Target Identification

Based on the existing understanding of the interactions between miRNAs and target mRNAs, various software tools have been developed to predict endogenous miRNA targets for experimental validation [95]. Recently, Ab Mutalib and colleagues reviewed the thirty-nine computational tools currently available for miRNA target prediction [96], of which only one, DeAnnIso, allows for target prediction of isomiRs [97]. These bioinformatic prediction tools are limited as they cannot predict miRNA binding to non-coding RNAs, nor do they account for non-canonical mRNA binding sites [98]. In addition, bioinformatics methods may give false-positive results, and miRNA target predictions do not always account for the tissue specificity of miRNA expression [99]; therefore, computational target predictions should always be validated experimentally.

The various experimental methods available for miRNA target validation in biological systems have been comprehensively reviewed elsewhere [100,101,102]. They include indirect methods such as expression profiling or stable isotope labeling by amino acids in cell culture (SILAC) following miRNA overexpression or inhibition, as well as direct methods such as reporter assays, biotinylated miRNA pulldown assays, and RISC component pulldown assays. In reporter assays, direct evidence of miRNA regulation is established when a mutated mRNA target site results in loss of miRNA regulation. In these experimental approaches, miRNAs are often overexpressed to supraphysiological levels, resulting in the saturation of RISC complexes at the expense of other endogenous miRNAs, and false-positive results that allow low-affinity targets to appear functionally relevant [103]. Nevertheless, these low-affinity targets, while irrelevant to the endogenous functioning of the miRNA in question, continue to be important when considering the cellular effects of miRNAs as potential therapeutics. Caution must also be exercised when extrapolating results from miRNA target validation experiments in particular cellular environments across tissue types, as the failure to detect cell-specific natural targets may ensue [100].

## 7. Functional miRNA Target Relationships in ACC

In ACC, the evidence for miRNA functional targets comes largely from reporter assays in combination with the cellular effects of modulation of miRNA expression in cell culture.

### 7.1. Overexpressed miRNAs and Their ACCs

Several miRNAs that are overexpressed in ACC relative to NAC have proven oncogenic roles *in vitro*, as well as defined molecular targets, that they regulate (Table 3). *miR-9* [104], *miR-21* [105], *miR-483-3p* [106], and *miR-483-5p* [106] have been well described in the literature as ‘oncomiRs’ across multiple mammalian cell types, which is consistent with their role in ACC. In contrast, *miR-139-5p* is an established tumor suppressor in head and neck/oral, breast, and gastric cancers [107], but is overexpressed in aggressive ACCs compared with non-aggressive ACCs [108].

#### 7.1.1. miR-9 Regulates LIN28

*miR-9* has diverse actions in cancer, and whether it acts as a tumor suppressor or oncomiR is tissue dependent [118]. In ACC, aggressive phenotypes overexpress *miR-9* in comparison with non-aggressive phenotypes [51], and *miR-9* overexpression is associated with poor prognosis in clinical datasets [93]. Luciferase-based assays have demonstrated direct binding between *miR-9* and LIN28, an RNA binding protein that regulates miRNA biogenesis via the miRNA *let-7* in HeLa and A2780 ovarian carcinoma cell lines [111]. Aggressive ACCs have been demonstrated to have weak LIN28 protein expression on immunostaining [93], lending evidence to the hypothesis that LIN28 expression is regulated by *miR-9* in this cellular environment.

#### 7.1.2. MiR-21 Regulates PDCD4

*MiR-21* is the most commonly overexpressed miRNA in cancer and is generally associated with an aggressive phenotype and poor prognosis. *miR-21* expression is negatively correlated with *Programmed Cell Death Protein 4* (*PCDC4*) expression across many solid tumors [105]. *PCDC4* is upregulated during apoptosis and inhibits translation of particular genes, including *p53*, by competitively binding translation initiation factors [119]. Luciferase reporter assays in HeLa cells as well as colorectal and thyroid cell lines, have established *miR-21* as a direct regulator of *PCDC4* expression [114,115]. In ACC, in vitro gene-specific silencing of *miR-21* resulted in increased *PCDC4* expression and reduced cellular proliferation [113], which suggests that this regulatory relationship between *miR-21* and *PCDC4* is also present in ACC.

#### 7.1.3. miR-483-3p Regulates PUMA

*miR-483-3p* is an oncomiR in ACC, promoting cellular proliferation and inhibiting apoptosis in in vitro cell models [74]. Reporter assays in three different cell lines, including human embryonic kidney (HEK293), liver cancer (HepG2), and colon cancer (HCT116), demonstrated that *miR-483-3p* directly inhibits *PUMA* expression [117]. *PUMA* is a downstream target of p53, which antagonizes the anti-apoptotic B-cell lymphoma 2 (Bcl-2) family proteins and consequently induces apoptosis [120]. *PUMA* expression was found to be inversely correlated with *miR-483-3p* expression in ACC, but not in ACA or NAC tissue [74]. Given that *miR-483-3p* is a proven regulator of *PUMA* expression in various cell models, this relationship can be extrapolated to ACC.

### 7.2. Underexpressed miRNAs and Their ACC Targets

The molecular targets of a number of underexpressed tumor suppressor miRNAs in ACC have also been characterized (Table 4). These miRNAs include *miR-7* and *miR-205* which have demonstrated tumor suppressor activity in in vivo xenograft ACC models [121,122]. They also include *miR-195* and *miR-497*, which are members of the tumor suppressor *miR-15* family and share the same seed sequence [123], as well as *miR-99* family members *miR-99a* and *miR-100*, which are known to target the mTOR signaling pathway [124].

#### 7.2.1. *miR-7* Regulates *Raf-1, EGFR, CDK1, PAK1, CKS2*

In the human genome, *miR-7* is encoded on three separate loci whose different DNA sequences can all be processed into the same mature *miR-7* sequence [130]. *miR-7* is an almost ubiquitous tumor suppressor, being underexpressed in malignancies that range from those derived from brain tissue to a myriad of solid tumors as well as leukemias [131]. In ACC, in vitro overexpression of *miR-7* decreases proliferation and induces G1 cell cycle arrest and decreases the expression of *p21 activated kinase 1* (*PAK1*), *CDC28 protein kinase regulatory subunit 2* (*CKS2*), and *cyclin-dependent kinase 1* (*CDK1*) mRNA [121]. PAK1 activation induces apoptosis, while CKS2 is an essential co-factor for CDK proteins that regulate the cell cycle. Luciferase reporter assays in H295R cells demonstrated the regulatory relationship between *miR-7* and *rapidly accelerated fibrosarcoma-1* (*Raf-1*) as well as the *epidermal growth factor receptor* (*EGFR*) [121]. *miR-7* targeting of *EGFR* has also been demonstrated in breast, lung, gastric, and ovarian cancers, as well as glioma and schwannoma tumors. In schwannomas and breast cancer, *miR-7* has also been shown to target *PAK1* [131].

#### 7.2.2. miR-99a/100 Regulates IGFR1, mTOR

Both *miR-99a* and *miR-100* were discovered as underexpressed relative to NAC in childhood ACC tissue samples, where their expression was inversely correlated with both *mTOR* and *insulin-like growth factor 1 receptor* (*IGFR1*) mRNA. *miR-100* specific knockdown in in vitro ACC cell models was associated with increased mTOR and IGFR1 protein expression, and furthermore, luciferase reporter assays in HEK293 cells showed that both miRNAs could regulate mTOR and IGFR1 [125]. The regulatory relationship between the *miR-99* family and mTOR has been well studied in cardiovascular disease [132] as well as in wound healing [133]. In cancer, *miR-99* regulation of mTOR has been demonstrated to enhance radiation sensitivity in urothelial carcinoma [134] as well as non-small cell lung cancer [135].

#### 7.2.3. miR-205 Regulates Bcl-2

In ACC, *miR-205* was shown to be underexpressed in a clinical cohort with RT-q-PCR. The subsequent gain of function studies carried out using SW13 cells (a cell line derived from adrenocortical metastasis of unknown origin) showed that *miR-205* promoted apoptosis and impaired cellular proliferation *in vitro*, and in vivo mouse SW13 xenograft studies showed that it could inhibit tumor growth [122]. Luciferase reporter assays also carried out in SW13 demonstrated a direct regulatory relationship between *miR-205* and B-cell lymphoma 2 (Bcl-2) protein, which is known to regulate the intrinsic apoptotic pathway in cancer [136].

#### 7.2.4. *miR-375* Regulates MTDH

*miR-375*, a known tumor suppressor in multiple cancers, is underexpressed in ACC [78], and in aldosterone-producing adrenal adenomas, its expression is correlated with the tumor size [127]. In vitro overexpression of *miR-375* reduces cellular proliferation and suppresses metadherin (MTDH), which functions to promote tumor invasion, metastasis, and chemoresistance. It also acts via the PI3K/Akt and Wnt/β-catenin pathways to promote cellular proliferation, invasion, and survival [137]. Luciferase reporter assays in H295R cells show that *miR-375* directly binds *MTDH* mRNA and regulates its expression in vitro in ACC.

#### 7.2.5. miR-431 Regulates ZEB1

In ACC clinical samples, *miR-431* is differentially expressed in chemosensitive tumors compared with chemoresistant tumors. The gain of function studies in H295R and primary ACC cells showed that *miR-431* overexpression decreased the IC50 of both doxorubicin and mitotane to inhibit cellular proliferation. In cells treated with doxorubicin, *miR-431* reversed epithelial-to-mesenchymal transition (EMT) [128]. Zinc finger E-box binding homeobox 1 (ZEB1), a protein that induces EMT in cancer cells, had already been established as a direct target of *miR-431* in hepatocellular carcinoma [138]. Both *ZEB1* mRNA and protein expression decreased in doxorubicin treated H295R cells overexpressing *miR-431* [128], indicating that this regulatory relationship between *miR-431* and ZEB1 is active in ACC.

#### 7.2.6. miR-497 Regulates TARBP2, DICER1, MALAT1, eIF4E, SFPQ

*miR-497* expression is dysregulated in many solid organ tumors, which suggests that it may play an important tumor suppressor role. Multiple studies have confirmed *miR-497* underexpression in ACC and its genomic location in a region of frequent LOH (17p13.1-13.3), in close proximity to the *p53* locus, indicates that it may play a role in ACC tumorigenesis [15]. In vitro H295R gain of function studies have shown that *miR-497* decreases cellular proliferation, increases apoptosis, and also induces G1 cell cycle arrest [74,129]. *miR-497* has been shown to directly target the miRNA biogenesis related proteins DICER1 and TARBP2 in Ago-2 IP assays, along with *miR-195*. This was confirmed with gain of function studies that showed an inverse correlation between *miR-497* expression and *DICER1*, as well as *TARBP2* mRNA and protein expression in H295R cells [58]. In a separate study, which was the first to demonstrate miRNA targeting of long non-coding RNAs in ACC, luciferase reporter assays demonstrated that *miR-497* regulates the expression of *metastasis-associated lung adenocarcinoma transcript 1* (*MALAT1*) [129]. *MALAT1* is overexpressed in numerous types of tumors, including ACC, and is known to promote cellular proliferation, apoptosis, migration, and invasion [138]. In H295R, *miR-497* overexpression and *MALAT1* knockdown inhibit the expression of *eukaryotic translation initiation factor 4E* (*eIF4E*), which directs ribosomes to the cap structure of mRNAs and is, therefore, essential for protein synthesis [129]. *miR-497* gain of function and *MALAT1* knockdown studies further demonstrated the reciprocal inhibitory relationship between them in in vitro ACC models.

Within the limits of the caveats previously outlined, we can infer from the above studies that miRNAs modulate many protein targets that are involved in key driver pathways in ACC.

## 8. miRNA Modulation of ACC Driver Pathways

A significant proportion of the identified ACC miRNA molecular targets play various roles in the established ACC driver pathways. This supports the notion that miRNA modulation of protein expression, which in healthy cells helps to finetune and maintain the homeostatic balance, can potentiate oncogenesis when dysregulated.

### 8.1. miRNA Modulators of the p53 Pathway in ACC

**Figure 1:**
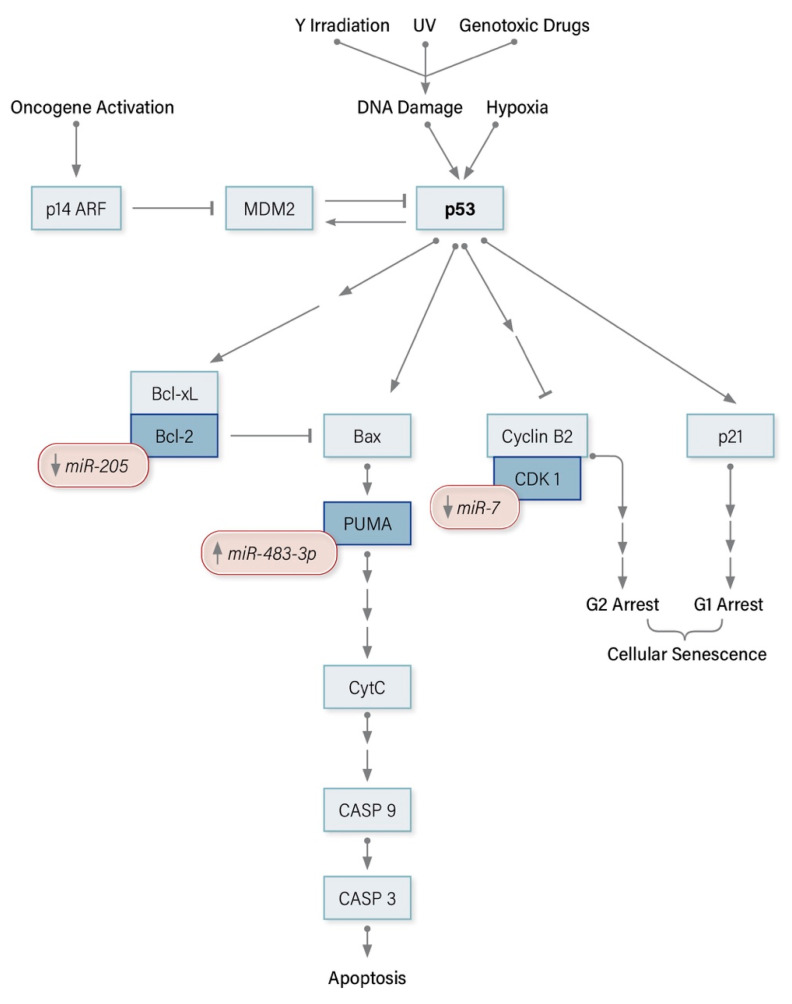
miRNA modulators of the p53 signaling pathway in Adrenocortical Carcinoma.

The overexpressed oncomiR *miR-483-3p* and the underexpressed tumor suppressors *miR-7* and *miR-205* all regulate downstream targets of p53 (Figure 1). *miR-483-3p* suppression of PUMA expression and the alleviation of *miR-205* modulated Bcl-2 inhibition of Bax, act synergistically to inhibit p53-mediated apoptosis. P53 is known to transcriptionally downregulate CDK1, and thus, initiates G2 cell cycle arrest. Constitutive activation of CDK1 resulting from the loss of *miR-7* targeted suppression overrides p53 mediated G2 arrest, leading to uncontrolled proliferation.

### 8.2. miRNA Modulators of the mTOR Pathway in ACC

**Figure 2:**
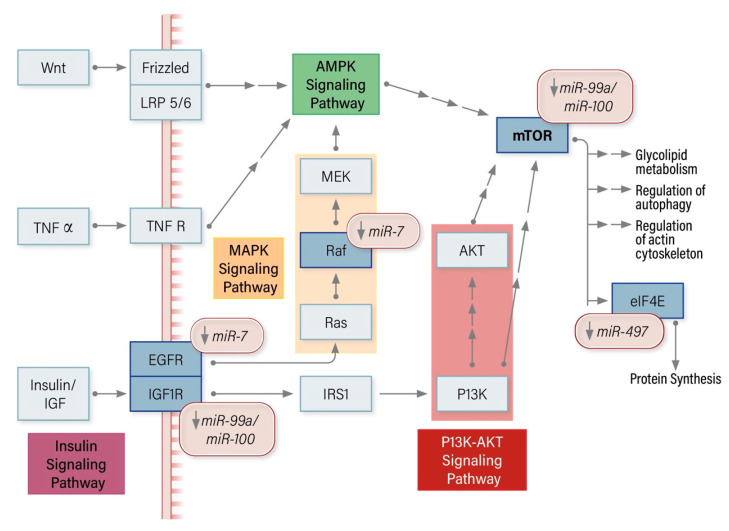
miRNA modulators of the mTOR signaling pathway in Adrenocortical Carcinoma.

The loss of *miR-7* regulation of Raf-1 and EGFR expression leads to downstream mTOR activation in ACC. The underexpression of *miR-99a/100* also leads to mTOR activation, ultimately potentiating protein synthesis, which is further enhanced by the loss of *miR-497* mediated eIF4E regulation. The loss of *miR-99a/100* mediated IGFR1 expression also promotes cell survival via the PI3K/AKT signaling pathway (Figure 2).

### 8.3. miRNA Modulators of the Wnt/Β-Catenin Pathway in ACC

**Figure 3:**
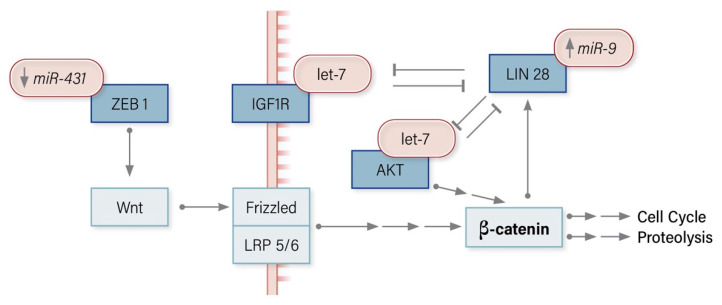
miRNA modulators of the Wnt/β-catenin signaling pathway in Adrenocortical Carcinoma.

In ACC, the loss of *miR-431* regulation allows ZEB1 to activate Wnt, consequently activating β-catenin, which potentiates cell cycling. The loss of *miR-375* mediated MTDH suppression upstream of MAPK modulates the Wnt/β-catenin pathway to promote cell cycling (Figure 3).

## 9. Future Directions and Conclusions

miRNAs play an important role in the modulation of ACC related target protein expression. The dysregulation of miRNA expression disturbs the homeostatic balance of proteins that participate in the pathways controlling cell cycle progression, cellular proliferation, apoptosis, and chemoresistance. The overexpression of oncogenic miRNAs and underexpression of tumor suppressor miRNAs thus potentiate tumorigenesis. The role of miRNA regulation in ACC remains an area of active research with the potential to further our understanding of its tumor biology and the molecular pathways involved. Small RNA sequencing of isomiRs and further refining our understanding of the miRNA signature of ACC provides the opportunity to improve diagnostic accuracy with techniques such as miRNA liquid biopsy. With continuing advances in functional techniques that allow molecular interactions to be clearly established, it will be possible to explore novel miRNA-based therapeutic approaches with the aim of improving the current poor prognosis of these patients.

## Figures and Tables

**Table 1:** Genetic syndromes associated with Adrenocortical Carcinoma.

Genetic Syndrome	Inheritance	Mutated Gene/s	Cellular Pathway/s Affected	Gene Locus	ACC Penetrance	Reference/s
Li Fraumeni Syndrome	Autosomal Dominant	*TP53*	Cell cycle	17p13.1	10%	[14]
Beckwith-Weidemann Syndrome	Sporadic	IGF2/H19 * CDKN1C/KCNQ1OT1 *	PI3K/TGF-β	11p15	7%	[16]
Familial Adenomatous Polyposis	Autosomal Dominant	*APC*	Wnt/β-Catenin	5q22.2	3%	[18]
Multiple Endocrine Neoplasia Type 1	Autosomal Dominant	*MEN1*	Cell Cycle	11q13	1–5%	[21,22]
Lynch Syndrome	Autosomal Dominant	*MLH1* *MSH2* *MSH6* *PMS2*	DNA mismatch repair	3p22.2 2p21 2p16.3 7p22	14 case reports	[23]
Neurofibromatosis Type 1	Autosomal Dominant	*NF1*	MAPK/ERK	17q11.2	9 case reports	[24]
Carney Complex	Autosomal Dominant	*CNC1 (PRKAR1A)*	cAMP	17q22-24	2 case reports	[25]

* Epigenetic modifications to methylation of imprinting control regions or paternal uniparental disomy are more common than gene mutations. IGF2, Insulin-like growth factor 2; CDKN1C, Cyclin-dependent kinase inhibitor 1C; KCNQ1OT, Potassium voltage-gated channel subfamily Q member 1 antisense gene; APC, Adenomatous polyposis coli; MEN1, Multiple endocrine neoplasia Type 1; MLH1, MutL homolog 1; MutS homolog 2; MSH6, MutS homolog 6; PMS2, PMS1 homolog 2; NF1, Neurofibromatosis 1; CNC1, Carney complex type 1; PRKAR1A, Protein kinase A regulatory subunit 1-alpha; PI3K, Phosphatidylinositol-3-kinase; TGF-β, Transforming growth factor-beta; Wnt, Wingless-related integration site; MAPK, Mitogen-activated protein kinase; ERK, Extracellular signal-related kinase; cAMP, cyclic adenosine monophosphate.

**Table 2:** Studies investigating differential expression of microRNAs in ACC Tissues compared with ACA and NAC.

Year of Publication	Methodology	Tissue Samples	Upregulated miRNA		Downregulated miRNA		Reference
2009	TLDA	7 ACC, 19 ACA, 10 NAC	*miR-184* *miR-210* *miR-503*		*miR-214* *miR-511* *miR-375*		[78]
2009	Microarray VC: RT-q-PCR	22 ACC, 27 ACA, 6 NAC VC (10 ACC, 9 ACA)	*miR-483-5p* *miR-503*		*miR-7* *miR-195*	*miR-335*	[73]
2011	Microarray VC: RT-q-PCR	25 ACC, 43 ACA, 10 NAC	*miR-483-3p* *miR-483-5p*	*miR-210* *miR-21*	*miR-195* *miR-497*		[74]
2011	Microarray VC: RT-q-PCR	10 ACC, 26 ACA VC (31 ACC, 35 ACA, 21 NAC)	*miR-483-5p*		*miR-195* *miR-125b*	*miR-100*	[75]
2011	TLDA VC: RT-q-PCR	7 ACC, 9 ACA, 4 NAC VC (16 ACC)	*miR-139-5p*		*miR-139-3p* *miR-675*	*miR-335*	[79]
2013	Microarray	12 ACC, 6 NAC VC (18 ACC, 10 ACA, 3 NAC)	*miR-483-5p* *miR-503* *miR-210* *miR-542-5p*	*miR-320a* *miR-93* *miR-148b*	*miR-195* *miR-335* *miR-497*	*miR-199a-5p* *miR-199a-3p*	[76]
2014	RT-q-PCR	51 ACC, 47 ACA	*miR-483-3p* *miR-483-5p* *miR-210*		*miR-195*		[80]
2014	RNA sequencing	45 ACC, 3 NAC	*miR-34b-5p* *miR-410* *miR-483-3p* *miR-483-5p* *miR-503*	*miR-506-3p* *miR-506-5p* *miR-508-3p* *miR-508-5p* *miR-510*	*miR-511* *miR-214-3p* *miR-485-3p* *miR-497* *miR-195*		[51]
2015	Microarray VC: RT-q-PCR	8 ACC, 25 ACA VC (11 ACC, 4 ACA)	*miR-503*		*miR-34a*	*miR-497*	[77]
2016	RNA sequencing	79 ACC, 120 NAC	*miR-10-5p* *miR-483-5p* *miR-22-3p* *miR-508-3p* *miR-509-3p*	*miR-509-5p* *miR-340* *miR-146a* *miR-21-3p* *miR-21-5p*			[81]
2017	RNA sequencing	7 ACC, 8 ACA, 8 NAC VC (8 ACC, 10 ACA, 10 NAC)	*miR-503-5p* *miR-450a-5p* *miR-542-5p* *miR-483-3p* *miR-542-3p* *miR-450b-5p* *miR-210* *miR-483-5p*	*miR-421* *miR-424-3p* *miR-424-5p* *miR-598* *miR-148b-3p* *miR-184* *miR-128*			[72]

TLDA, TaqMan Low Density Array; VC, Validation Cohort; ACC, adrenocortical carcinoma; ACA, adrenal adenoma; NAC, normal adrenal cortex.

**Table 3:** Overexpressed microRNAs and their regulated targets in ACC.

microRNA	Functional Role in ACC	Molecular Target	Evidence for Regulatory Interaction	Reference/s
*miR-9*	Associated with reduced DFS and increased recurrence in clinical data	*LIN28*	Weak protein expression pattern in aggressive ACC Established reporter assays in non-ACC models	[109,110,111]
*miR-21*	↑ cellular proliferation	*PCDC4*	Inverse correlation in expression Reporter assays in non-ACC cell models	[112,113,114,115]
*miR-139-5p*	Associated with anchorage-independent colony formation	*NDRG4*	Inverse correlation in expression Reporter assays in non-ACC cell models	[116]
*miR-483-3p*	↑ cellular proliferation ↓ apoptosis	*PUMA*	Inverse correlation in expression Reporter assays in non-ACC models	[74,117]
*miR-483-5p*	Associated with anchorage-independent colony formation	*NDRG2*	Inverse correlation in expression Reporter assays in non-ACC models	[116]

DFS, Disease-Free Survival; PDCD4, Programmed cell death protein 4; NDRG, N-myc downstream-regulated gene; PUMA, p53 upregulated modulator of apoptosis.

**Table 4:** Underexpressed microRNAs and their regulated targets in ACC.

MicroRNA	Functional Role in ACC	Molecular Target	Evidence for Regulatory Interaction	Reference/s
*miR-7*	↓ cellular proliferation ↑ G1 cell cycle arrest ↓ H295R xenograft growth *in vivo*	Raf-1 * EGFR * *CDK1* *PAK1* *CKS2*	Reporter assays * Inverse correlation in expression	[121]
*miR-99a*		*IGFR1* *mTOR*	Inverse correlation in expression Reporter assays in non-ACC models	[125]
*miR-100*		*IGFR1* *mTOR*	Inverse correlation in expression Reporter assays in non-ACC models	[125]
*miR-195*	↓ cellular proliferation ↑ cellular invasion ↑ apoptosis	*TARBP2* *DICER1*	Inverse correlation in expression Ago-2 IP	[58,74]
		*ZNF367*	Reporter assays in SW13 cells	[126]
*miR-205*	↓ cellular proliferation ↑ apoptosis ↓ SW13 xenograft tumor growth *in vivo*	*Bcl-2*	Reporter assays	[122]
*miR-375*	↓ cellular proliferation	*MTDH*	Reporter assays Inverse correlation in expression	[127]
*miR-431*	↑ cellular sensitivity to doxorubicin and mitotane	*ZEB1*	Inverse correlation in expression in miRNA overexpressing doxorubicin treated H295R cells. Reporter assays in non-ACC models	[128]
*miR-497*	↓ cellular proliferation ↑ apoptosis ↑ G1 cell cycle arrest	*TARBP2* *DICER1*	Inverse correlation in expressionAgo-2 IP	[58,74]
		*MALAT1* *eIF4E* *SFPQ*	Inverse correlation in expression Reporter assays	[129]

* Regulatory interaction demonstrated by reporter assays for Raf-1 and EGFR only. Raf-1, Rapidly Accelerated Fibrosarcoma-1; EGFR, Epidermal Growth Factor Receptor; PAK1, p21 activated kinase 1; CKS2, CDC28 Protein Kinase Regulatory Subunit 2; CDK1, Cyclin-Dependent Kinase 1; IGFR1, Insulin-like Growth Factor 1 Receptor; mTOR, Mechanistic Target of Rapamycin Kinase; TARBP2, TAR RNA-binding protein 2; DICER1, Dicer 1 Ribonuclease III; Ago-2 IP, Argonaute-2 immunoprecipitation; ZNF367, Zinc Finger Protein 367; Bcl-2, B-cell lymphoma 2; MTDH, metadherin; ZEB1, Zinc finger E-box binding homeobox 1; MALAT1, metastasis-associated lung adenocarcinoma transcript 1; eIF4E, eukaryotic translation initiation factor 4E; SFPQ, splicing factor proline and glutamine-rich.
